# 

**Published:** 1892-01

**Authors:** 


Whole Number, CCCLXIV.

New Series.

January, 1892.

VOL. XXXI. No. 6.

LIBRA[illegible] Index S. G. [illegible]

### Buffalo Medical and Surgical Journal.

Established 1845 by AUSTIN FLINT, M. D.

**Editors:**

THOS. LOTHROP, M. D. WM. WARREN POTTER, M. D.

**Associate Editors:**

WILLIAM C. KRAUSS, M. D. JAMES WRIGHT PUTNAM, M. D.

JOHN A. MILLER, Ph. D. DeLANCEY ROCHESTER, M. D.

JOHN PARMENTER, M. D.

SCRIPTA FERUNT ANNOS

European Agency,
TRÜBNER & CO.,
57 and 59 Ludgate Hill, London, E. C.

BUFFALO:
1891.

Foreign Subscription, $2.50

Entered at the Post-Office at Buffalo, N. Y., as Second-class Mail Matter.

*Times Printing House, Buffalo, N. Y.*

SUBSCRIPTION PRICE, $2.00 IF PAID IN ADVANCE; OTHERWISE $2.50 A YEAR.

PUBLISHED MONTHLY.

Table of Contents on Page V.

